# Hydroxytyrosol attenuates intimal hyperplasia in *in vitro* and *ex vivo* human saphenous vein models: association with Akt and Nrf2/HO-1 signalling

**DOI:** 10.3389/fphar.2026.1962884

**Published:** 2026-10-28

**Authors:** Ubashini Vijakumaran, Muhammad Dain Yazid, Eva Jover, Nur Ayub Md Ali, Hairulfaizi Haron, Muhammad Ishamuddin Ismail, Mohd Ramzisham Abdul Rahman, Nadiah Sulaiman

**Affiliations:** 1 Department of Tissue Engineering and Regenerative Medicine, Faculty of Medicine, Universiti Kebangsaan Malaysia, Kuala Lumpur, Malaysia; 2 Institute of Medical Science Technology, Universiti Kuala Lumpur (UniKL), Kajang, Selangor, Malaysia; 3 Cardiovascular and Pulmonary (CardioResp) Research Group, Universiti Kebangsaan Malaysia, Bangi, Selangor, Malaysia; 4 Cardiovascular Translational Research. Navarrabiomed (Fundación Miguel Servet), Instituto de Investigación Sanitaria de Navarra (IdiSNA), Hospital Universitario de Navarra (HUN), Universidad Pública de Navarra (UPNA), Pamplona, Spain; 5 Pusat Jantung Dan Paru-Paru, Hospital Canselor Tuanku Muhriz, Jalan Yaacob Latif, Bandar Tun Razak, Kuala Lumpur, Malaysia; 6 Department of Surgery, Faculty of Medicine, Universiti Kebangsaan Malaysia, Kuala Lumpur, Malaysia

**Keywords:** endothelial cells, hydroxytyrosol, saphenous vein graft, vascular smooth muscle cells, venous intimal hyperplasia

## Abstract

**Background:**

Venous intimal hyperplasia remains a major cause of saphenous vein graft failure following coronary artery bypass grafting Current antiproliferative strategies often impair endothelial cell function, exacerbating vascular injury. Hydroxytyrosol (HT), a bioactive olive phenolic compound, exhibits antioxidant and anti-inflammatory properties, but its coordinated effects on vascular smooth muscle cells and ECs remain unclear.

**Methods:**

Primary human saphenous vein-derived SMCs and ECs were exposed to graded concentrations of HT to evaluate cell viability, proliferation, migration, chemotaxis, and signalling responses. A human saphenous vein ring (hSVR) culture model was used to investigate the effects of HT on spontaneous *ex vivo* intimal hyperplasia.

**Results:**

HT (20–320 μM) significantly inhibited PDGF-BB-induced SMC proliferation, migration, and chemotaxis accompanied by reduced Akt signalling. In contrast, HT at concentrations ≤50 μM did not produce a statistically significant reduction in EC viability, proliferation, and migration while activating the Nrf2/HO-1 antioxidant pathway. In the *ex vivo* hSVR model, 50 μM HT reduced intimal thickness by 45.2% and significantly attenuated neointimal cell proliferation at day 21. HT also suppressed Akt activation, enhanced HO-1 expression, and reduced vimentin expression, consistent with attenuation of vascular remodeling. Attempts to evaluate re-endothelialization were inconclusive because seeded ECs failed to establish continuous luminal coverage in the *ex vivo* model.

**Conclusion:**

Hydroxytyrosol selectively inhibits SMC activation while preserving endothelial homeostasis associated with differential modulation of the Akt and Nrf2/HO-1 pathways. These findings demonstrate that HT effectively attenuates intimal hyperplasia in a human *ex vivo* vein model and support its potential as an adjunctive strategy for improving vein graft patency. Further studies using perfused vascular systems or *in vivo* models are required to determine its effects on endothelial repair and re-endothelialisation.

## Graphical Abstract

**Figure:**
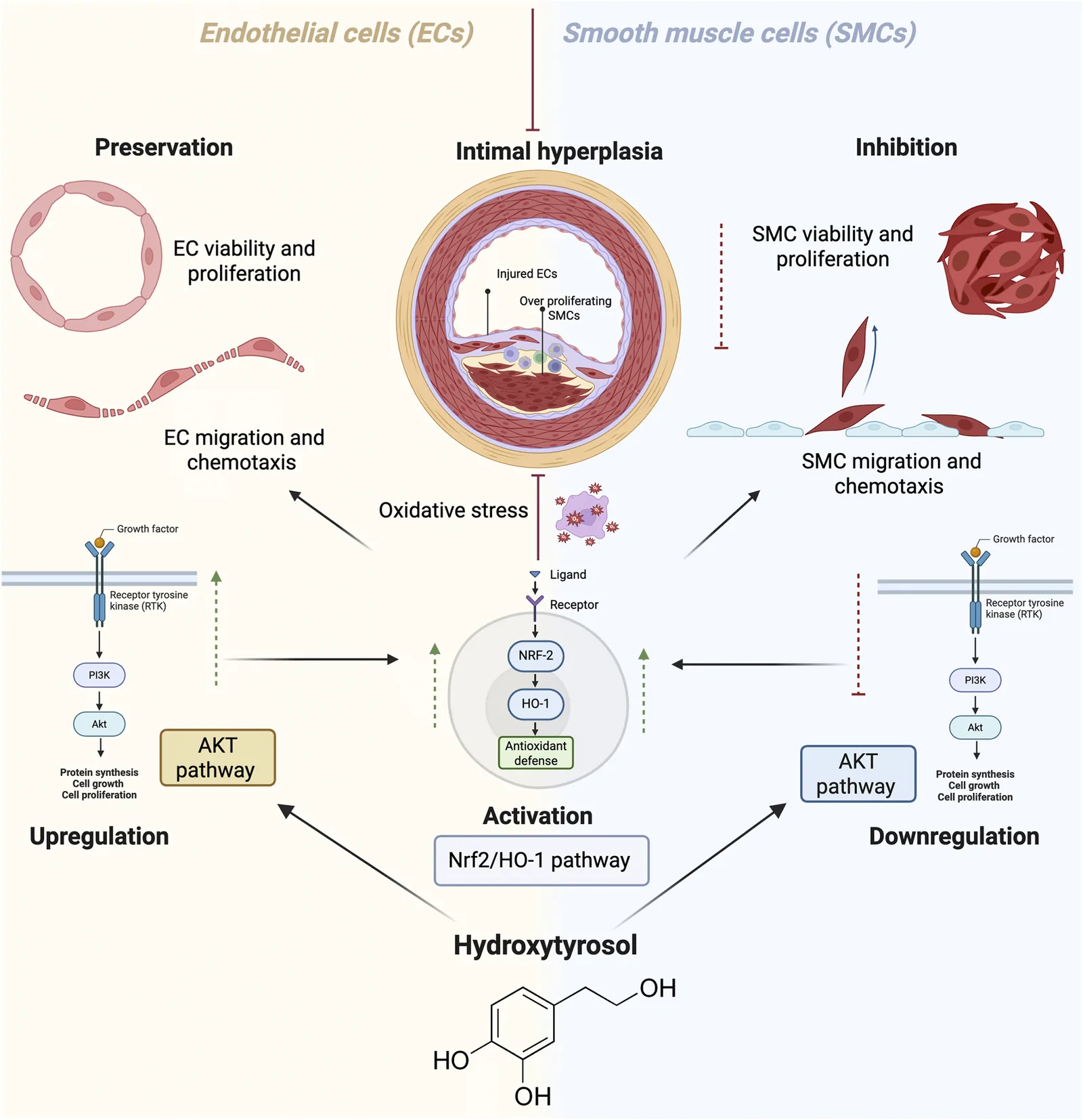

## 1 Introduction

Globally, an estimated 244.1 million people are afflicted with ischemic heart disease (IHD) (Feigin et al., 2022). The primary revascularization techniques for IHD are percutaneous coronary intervention (PCI) and coronary artery bypass grafting (CABG). Although arterial conduits such as the internal thoracic artery and radial artery are increasingly favored in contemporary CABG due to superior long-term patency (Caliskan et al., 2020), saphenous vein grafts (SVGs) continue to constitute a substantial proportion of grafts used worldwide. Among the available conduits, the saphenous vein remains favored because of its relative ease of harvest and sufficient length, allowing multiple distal anastomoses, particularly in patients requiring multi-vessel revascularization (Caliskan et al., 2020; Altshuler et al., 2025). Contemporary registry data further confirm that SVGs remain widely utilized in real-world CABG practice, especially when arterial conduits are limited or insufficient (Kim et al., 2023). However, the long-term viability of saphenous vein grafts is limited, with a patency rate of only 50% after 10 years (de Vries and Quax, 2018). Therefore, understanding the mechanisms underlying SVG failure remains clinically relevant despite evolving surgical strategies.

A significant factor contributing to the decline in graft patency is intimal hyperplasia (IH), a vascular condition characterized by the thickening of the intima layer with subsequent narrowing of the vessel lumen (Gellman et al., 1991; Cornelissen et al., 2022). Endovascular surgical procedures often result in endothelial damage (Modi et al., 2024) which triggers a cascade of biological responses that activates platelets and vascular smooth muscle cells (SMCs). Activated platelets release growth factors such as platelet-derived growth factor (PDGF) and thromboxane, which drive excessive proliferation and migration of SMCs into the intimal layer, ultimately accelerating the atherosclerotic process (Zeng et al., 2019; Zhang et al., 2022).

Nevertheless, millions of drug-eluting stents are implanted worldwide every year. Advancements in second- and third generation DES (G2/G3-DES), including improved flexibility, enhanced reendothelialization, reduced strut thickness, and optimized drug elution have made G2/G3-DES the predominant choice for angioplasty (Diaz-Rodriguez et al., 2021; Cornelissen et al., 2022; Cutlip et al., 2022). Despite these improvements, large-scale registries continue to report cases of stent thrombosis, (Kuramitsu et al., 2021), accounting for an annual global revascularisation rate of 1%–2% (Giustino et al., 2022). Additionally, genetic mutations that confer resistance to coated drugs such as rapamycin (Huang and Houghton, 2001; Aoki and Tanabe, 2021) may further contribute to persistent complications observed. Jana’s review highlighted strategies to modify prosthetic devices, including their surface to release nitric oxide (NO) and biomolecules to promote reendothelisation (Jana, 2019). Consequently, plant-based adjuvants have gained attention as potential alternatives to prevent restenosis. Natural compounds such as Andrographolide (Han et al., 2023), Farrerol (Fang et al., 2023), Bunao-Fuyuan decoction (Guo et al., 2021), myricanol (Fan et al., 2021), and Glossogyne tenuifolia (Hsuan et al., 2020), have been shown to inhibit SMC’s proliferation and migration. On the other hand, resveratrol has demonstrated positive effects on EC, underscoring the relative lack of research on EC responses compared to the SMC (Zhao and Feng, 2020). Most existing studies are more focused on SMC inhibition, with limited evaluation of the compound effects on ECs. This gap highlights the need for further research into the impact of these compounds on endothelial function, given its critical role in early pathogenesis of IH.

HT is a naturally occurring phenolic compound in olive oil, known for its cardioprotective properties, including LDL oxidation inhibition, reduced platelet aggregation, and anti-inflammatory effects in atherosclerosis development (Vijakumaran et al., 2023a; Noguera-Navarro et al., 2023; Vijakumaran et al., 2023b). While previous studies focused on these benefits, research on its concurrent effects on both SMCs and ECs *in vitro* or *ex vivo* remain limited. Given that most studies on restenosis prevention primarily target SMC inhibition without considering the impact on EC function, this represents an unmet critical research gap. Maintaining endothelial integrity is essential for vascular homeostasis, yet little is known about how HT influences both vascular cell types within the same physiological environment. Therefore, the present study aimed to (1) identify an optimal HT dose that inhibits mitogen SMCs activation while preserving the EC function, using primary cell culture derived from patients who may benefit from anti-restenosis strategies; (2) investigate the mechanisms involved in such effects; and (3) develop a viable *ex vivo* model to assess HT and/or other naturally derived compounds, as preclinical steps towards clinical applications.

## 2 Methodology

### 2.1 Preparation of hydroxytyrosol

3-Hydroxytyrosol (MW: 154.16g) (HT) was purchased from Sigma, United States with a purity exceeding 98%, as verified by High-performance liquid chromatography (HPLC). The lyophilized powder was dissolved in sterile phosphate buffer saline (PBS) to prepare a 1 mM stock solution, which was subsequently diluted in cell media to achieve working concentrations ranging from 6.25 µM to 320 µM.

### 2.2 Patient recruitment and sample collection

This study utilized surplus human saphenous vein (hSV) segments from patients undergoing elective coronary artery bypass grafting (CABG) at Hospital Canselor Tuanku Muhriz (HCTM). Ethical approval was obtained from the Research Ethics Committee of Universiti Kebangsaan Malaysia UKM/PPI/111/8/JEP-2020-521 ensuring compliance with Malaysian law and the ethical principles outlined in the 1975 Declaration of Helsinki and its subsequent amendments. All adult patients undergoing CABG were included regardless of age, except those with malignant tumor, chronic inflammatory diseases, and infectious diseases. Written informed consent was obtained from all participants. Detailed donor demographic and clinical characteristics, including age, sex, cardiovascular risk factors, and medication status, of the CABG cohort used for primary cell isolation have been previously reported (Haron et al., 2024). Briefly, the cohort consisted of patients with a mean age of 59 ± 9.2 years, predominantly male (80%), with common comorbidities including hypertension, diabetes mellitus, hyperlipidemia, and smoking history given in Supplementary Table S1.

### 2.3 Human primary cell isolation and culture

Surplus hSV segments were collected post-surgery in Dulbecco’s PBS (DPBS) and immediately processed for ECs and SMC isolation. Primary SMCs and ECs were isolated from independent donor samples obtained from different patients undergoing CABG. Each biological replicate (N) represents cells derived from a distinct patient. Technical replicates (n) correspond to repeated measurements performed per donor within the same experimental condition. Primary SMCs and ECs were used between passages three and five for all experiments. SMCs and ECs used in the study were not necessarily derived from matched samples of the same individual. ECs were obtained using an explant culture method as described by Zhu et al. (2019), whereas SMCs were isolated following the protocol by Metz et al. (2012). Cells were maintained in specialized growth media: ECs in Endothelial Cell Growth Medium-2 (EGM-2; PromoCell, Germany) and SMCs in Smooth Muscle Cell Growth Medium-2 (SMC-2; PromoCell, Germany), following the manufacturer’s guidelines. Phenotypic confirmation was conducted via immunocytochemistry using CD31 for ECs and smooth muscle actin (SMA) for SMCs.

### 2.4 Cell viability/metabolic activity assay (MTT assay)

3-(4,5-dimethylthiazol-2-yl)-2,5-diphenyltetrazolium bromide, a tetrazole (MTT) (Sigma, United States) was employed to assess the optimal HT concentration for metabolic activity/cell viability. SMCs were used at 50% confluence. SMCs were serum-starved overnight, and subsequently 24 h-stimulated with 50 ng/mL of Platelet-derived growth factor-BB (PDGF-BB) (Lim et al., 2010; Li et al., 2011). After 24 h PDGF-BB stimulation, cells were washed and subsequently treated with HT (10 μM, 20 μM, 40 μM, 80 μM, 160 μM and 320 μM) for up to 48 h (Zrelli et al., 2011a; Abe et al., 2012) in fresh medium. On the other hand, ECs were seeded at 10,000 cells/well in 48 well plates (10,869 ECs/cm^2^), serum-starved for 4 h serum starved for 4 h and treated with HT (6.25 μM, 12.5 μM, 25 μM, 50 μM, 100 μM and 200 μM) (Abate et al., 2020) for up to 48 h. Following treatment, 20 μL of MTT reagent was added to each well with 200 μL of basal media and incubated for 4 h before measuring optical density (OD) at 540 nm. Untreated cells served as a positive control for cell viability. Viability was expressed as percentage relative to control

$$\left(OD\;of\;treatment/OD\;of\;control*100\right).$$

### 2.5 Cell proliferation assay (EdU assay)

Cell proliferation was assessed using the Click-iT EdU Alexa Fluor 488 Imaging Kit (C10337) (Thermofisher, United States). In brief, cells were co-treated with HT and 10 μM EdU for 48 h. Fixation was performed using 4%-buffered paraformaldehyde (PFA), followed by permeabilization with 0.25% Triton-X100. EdU incorporation was visualized using fluorescence microscopy (Nikon Eclipse Ti, Japan), with Hoechst (33,342) staining (300 nM) for nuclei. Pictures were taken from ten different fields. Green (proliferating cells) and blue (total cell count) colour-stained nuclei were calculated using ImageJ software. Untreated cells served as control. Lastly, the percentage of proliferation was calculated as

$$\left[Positive\;proliferation\frac{\left(green\;nuclei\right)}{\left(blue\;nuclei\right)}Total\;cell\;count\;\left(green\;and\;blue\;nuclei\right)\right]\;x\;100.$$

### 2.6 Scratch assay

Cell migration was assessed using a scratch assay using the modified protocol by Wang et al. (2022). Prior to scratching, cells were serum-starved to minimize basal proliferation and synchronize cell cycles. SMCs were cultured to ∼80% confluence and stimulated with PDGF-BB (50 ng/mL). After scratch induction using a sterile p10 pipette tip and DPBS washing, cells were treated with HT (20, 40, 80 µM) in serum-free medium containing PDGF-BB for 10 h. The 10-h endpoint was selected based on preliminary optimization, as PDGF stimulation markedly accelerates SMC migration and wound closure occurred before 24 h, limiting accurate quantification. ECs were seeded at 10,000 cells/cm^2^ and grown to full confluence. Following scratch induction and DPBS washing, cells were treated with HT (12.5, 25, 50, 100 µM) in serum-free medium for 24 h. Images were captured from six fixed fields per well at 0 h and at the respective endpoints using a Nikon Eclipse Ti inverted microscope. Wound area was quantified using ImageJ software and expressed as percentage gap closure:

$$\%GAP=\left[\left(initial\;area-final\;area\right)÷initial\;area\right]x\;100$$

### 2.7 Transwell migration assay

A Boyden chamber (Transwell) migration assay was performed to evaluate the effect of hydroxytyrosol (HT) on cell migration. Briefly, 25,000 cells suspended in 300 μL serum-free medium were seeded into the upper chamber of a Transwell insert (8 μm pore polycarbonate membrane, 1.12 cm^2^; Corning, United States). The lower chamber contained 700 μL of culture medium with or without hydroxytyrosol. For smooth muscle cells (SMCs), the lower chamber medium contained PDGF-BB (50 ng/mL) as a migratory stimulus, with or without HT (20, 40, or 80 μM). For endothelial cells (ECs), HT was added at concentrations of 12.5, 25, 50, or 100 μM. This setup established a PDGF-BB gradient across the membrane, allowing assessment of PDGF-BB–stimulated transwell migration. Cells were allowed to migrate for 10 h. Following incubation, inserts were gently washed with DPBS and non-migrated cells remaining on the upper surface of the membrane were removed using a sterile cotton swab. Cells that migrated to the underside of the membrane were then fixed with 4% buffered paraformaldehyde (PFA) for 2 min, washed twice with DPBS, and permeabilized with 100% methanol for 20 min. After washing with DPBS, migrated cells were stained with 0.5% crystal violet (Sigma, United States) for 20 min in the dark. Excess dye was removed by washing with DPBS, and the migrated cells on the underside of the membrane were visualized using a light microscope. Ten random microscopic fields per insert were captured, and crystal violet–stained migrated cells were segmented and quantified using ImageJ software. The area occupied by stained cells within each field was used as a relative indicator of migrated cell abundance.

### 2.8 Western blot analyses

Cells were lysed using RIPA buffer (Thermo Fisher Scientific, United States) supplemented with a protease inhibitor cocktail (Sigma, United States). The lysates were centrifuged at ∼14,000 × g for 15 min at 4 °C, and the supernatants were collected for protein expression analysis. Protein concentrations were determined using the bicinchoninic acid (BCA) assay (Thermo Fisher Scientific, United States). Equal amounts of protein (20 μg) were separated on 10% SDS–PAGE gels at 120 V for approximately 1 h and transferred onto nitrocellulose membranes using a wet transfer system (Invitrogen Mini Blot Module) at 10 V for 1 h. Membranes were blocked with 5% non-fat dried milk in TBST (0.1% Tween-20) for 1 h at room temperature. Primary antibodies were incubated overnight at 4 °C, with antibody solutions prepared in BSA-containing buffer to facilitate phospho-protein detection. Following primary antibody incubation, membranes were washed with TBST and incubated with HRP-conjugated secondary antibodies (Amersham™ ECL™ anti-rabbit or anti-mouse IgG HRP; 0.4 μg/mL; GENA934 or GENA931, respectively; Merck-Sigma) for 1 h at room temperature. Protein bands were visualized using an Amersham Imager 600, and exposure times were optimized to ensure signals remained within the linear detection range and did not reach saturation. Densitometric analysis was performed using ImageJ software. For Akt signaling analysis, phosphorylated Akt levels were quantified as the p-Akt/total Akt ratio. Details of antibody sources and dilution factors are provided in the Supplementary Table S2.

### 2.9 Saphenous vein ring culture

The procedure was adapted and modified from previous protocols (Schachner et al., 2007; Wong et al., 2016). hSV segments were washed with 5–10 mL DPBS. The adventitial layer was carefully removed using fine forceps prior to ring preparation, following the approach commonly used in human saphenous vein organ culture models to minimize adventitial fibroblast contamination and to focus on medial and intimal responses (Wilson et al., 1996). Vein segments were cut into 1 mm thick rings. Each ring was dried on a sterile filter and placed into 48-well plates thereafter. The rings were cultured in endothelial growth medium (EGM-2) with or without 50 μM HT with 1 μM EdU. EGM2 without HT served as a control. Vein rings were cultured until day 21. Media consisting of EGM-2 with 50 μM HT and 1 μM EdU was refreshed every 2 days. In the *ex vivo* vein ring culture model, EdU was maintained in the culture medium during the culture period to capture cumulative proliferative activity associated with neointimal remodelling. For protein expression analysis, three vein rings from technical replicates were pooled prior to protein extraction for Western blot analysis. Experimental design is illustrated in Figure 1.

**FIGURE 1:**
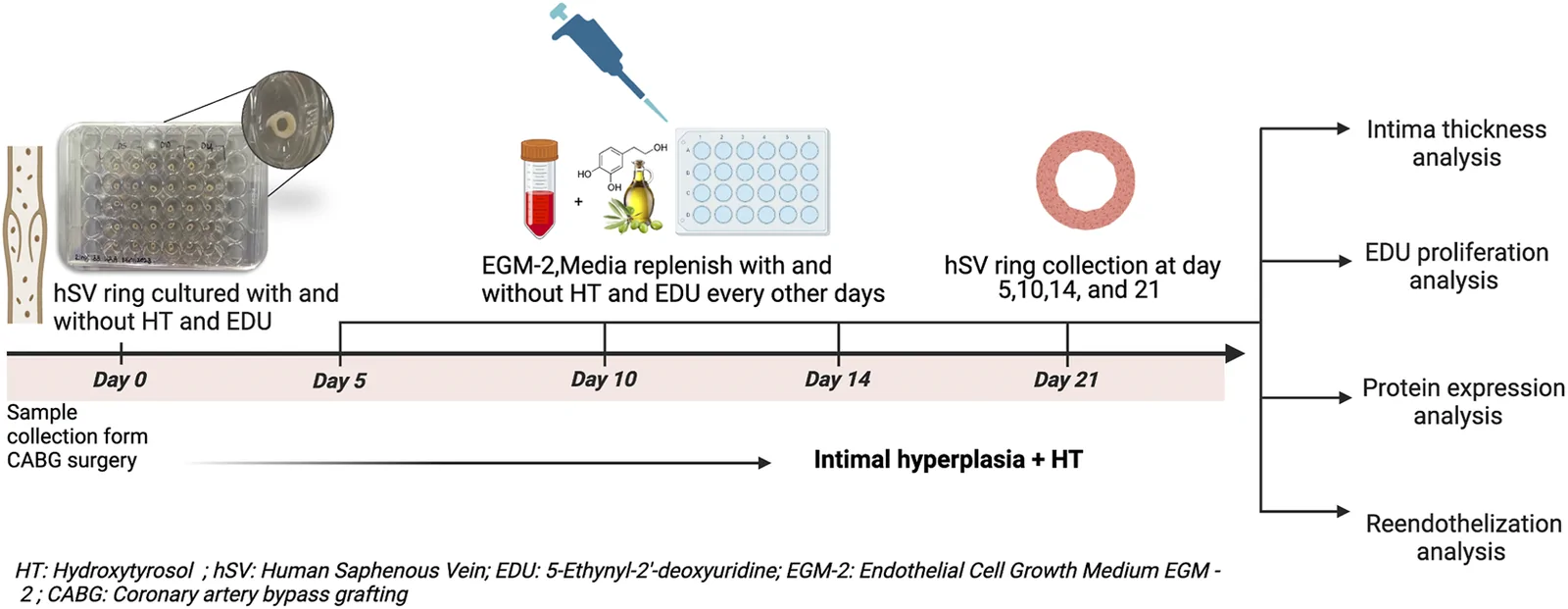
Experimental Design Scheme: Schematic illustration of the *ex vivo* human saphenous vein (hSV) ring culture. The hSV rings were divided into two groups: Group 1 was treated with 1 μM EdU and 50 μM HT, while Group 2 served as the control with untreated (no HT) media. Treatment or control untreated media were refreshed/replaced every 2 days, as appropriate. The hSV rings were collected at days 5, 10, 14, and 21. Samples (N = 6) were fixed in 10% formalin for subsequent histological analysis, while some other rings (N = 3) were lysed for protein expression studies.

### 2.10 Reendothelisation in saphenous vein rings

Saphenous vein segments were cut into approximately 1-mm-thick rings and allocated to three experimental groups: veins alone, veins + endothelial cells (ECs), and veins + ECs + hydroxytyrosol (HT). A total of 20,000 ECs was seeded onto each vein ring. During the initial seeding period, the rings were maintained under gentle rotational conditions to enhance cell–tissue contact, followed by static culture for the remainder of the experiment. Owing to the three-dimensional architecture and partial collapse of the vein rings during culture, exclusive luminal attachment of ECs could not be ensured. The rings were cultured in EGM-2 medium with or without 50 μM HT for up to 7 days. Samples were collected on days 5 and 7, fixed in 10% neutral-buffered formalin, and processed for immunofluorescence staining.

### 2.11 Assessment of intimal thickness and proliferation

Haematoxylin and eosin (H&E) staining, Masson’s Trichrome staining, immunofluorescence staining, and 5-ethynyl-2′-deoxyuridine (EdU) staining were performed to evaluate the effect of hydroxytyrosol (HT) on intimal hyperplasia in the *ex vivo* human saphenous vein ring (hSVR) model. Intimal morphology was visualized using H&E staining and imaged with an Olympus BX53 microscope. Intimal thickness was quantified using a radial morphometric approach. Briefly, the distance from the luminal surface to the internal elastic lamina (IEL) was measured at eight evenly distributed circumferential points per cross-section using ImageJ software. The mean of these measurements was calculated for each section. This approach was employed to minimise bias arising from variable luminal deformation inherent to *ex vivo*, non-pressurized vascular specimens. Histomorphometric analysis was performed blinded to treatment allocation, with images coded by a research assistant prior to quantification. Cell proliferation was assessed using the EdU incorporation assay. EdU-positive nuclei were quantified within the intimal layer and expressed relative to total nuclei in the same region. For protein expression analysis, tissue samples were lysed, and equal amounts of protein were subjected to Western blotting.

### 2.12 Statistical analysis

Statistical analyses were performed using GraphPad Prism version 9.5 (GraphPad Software, San Diego, CA, United States). For all assays, technical replicates were averaged to yield a single value per donor prior to statistical analysis; the donor mean, not individual technical replicates, was treated as the unit of biological replication (N). Data were assessed for normality using the Shapiro–Wilk test because of the small sample size. Where appropriate, data were log-transformed before statistical analysis to improve normality. Comparisons among multiple groups with a single independent variable were performed using one-way analysis of variance (ANOVA) followed by Dunnett’s multiple comparisons test. Comparisons involving two independent variables (treatment and time) were analyzed using two-way ANOVA, followed by either Dunnett’s or Šídák’s multiple comparisons test, as appropriate for the planned comparisons. A two-tailed p-value <0.05 was considered statistically significant. Data are presented as mean ± standard error of the mean (SEM).

## 3 Results

### 3.1 HT inhibited PDGF-BB-induced SMC metabolic activity/viability, proliferation, migration, and transwell migration

HT inhibited PDGF-BB-induced proliferative and migratory responses in primary-cultured SMCs in a concentration-dependent manner (Figure 2). For the proliferation assay, HT significantly reduced SMC proliferation at concentrations of 20–320 μM following both 24 h and 48 h of treatment (Figure 2A). PDGF-BB treatment increased SMC proliferation to 30.25% ± 2.34% at 48 h compared with the untreated control. This increase was markedly attenuated by HT, with proliferation reduced to 4.66% ± 1.23% (p < 0.0001) at the highest concentration tested (320 μM). For metabolic activity/cell viability, PDGF-BB treatment resulted in a non-significant increase at 24 h (114.17% ± 2.84%, p = 0.0968) and a significant increase at 48 h (121.70% ± 3.60%, p = 0.0025) compared with the untreated control (Figure 2B). At 24 h, HT significantly reduced PDGF-BB-induced viability at concentrations of 160–320 μM. At 48 h, significant reductions were observed at concentrations of 40–320 μM compared with the PDGF-BB-treated group. For example, treatment with 40 μM HT reduced metabolic activity/viability to 103.98% ± 4.28% (p = 0.0207), while 320 μM HT further reduced viability to 61.61% ± 7.91% (p < 0.0001). Significant inhibition of SMC proliferation was also observed from 20 μM HT onwards, with proliferation reduced to 20.28% ± 2.33% (p = 0.0006). To determine whether these reductions were attributable to reduced cell number rather than a specific antiproliferative effect, total nuclei (Hoechst-positive, EdU-positive and EdU-negative combined) were quantified across the same fields used for EdU analysis, across the full concentration range (10–320 μM) at both 24 h and 48 h, in N = 6 independent donors (Supplementary Figure S1). Total nuclei counts did not differ significantly between PDGF-BB alone and any HT-treated group, or the untreated control, at either timepoint (two-way ANOVA with Šídák’s multiple comparisons test; all adjusted p ≥ 0.44), indicating that the observed reductions in EdU incorporation and migration reflect an antiproliferative rather than cytotoxic effect. PDGF-BB significantly enhanced SMC migration in the scratch wound assay compared with the untreated control (49.34% ± 5.00% vs. 28.88% ± 1.35%, p = 0.0006; Figure 2D). HT treatment significantly reduced wound closure at all concentrations tested. Gap closure was reduced to 34.31% ± 3.90%, 29.58% ± 2.60%, and 24.03% ± 1.66% following treatment with 20, 40, and 80 μM HT, respectively. The effect of HT on directed SMC migration was further evaluated using a Boyden chamber assay (Figure 2E). PDGF-BB significantly increased migration to 184.79% ± 27.16% relative to the untreated control. HT attenuated this response in a concentration-dependent manner. Treatment with 20 μM HT reduced migration to 148.28% ± 18.51%, although this did not reach statistical significance (p = 0.3916). In contrast, significant reductions were observed at 40 μM (86.38% ± 12.74%, p = 0.0023) and 80 μM (81.19% ± 15.28%, p = 0.0014) compared with the PDGF-BB-treated group.

**FIGURE 2:**
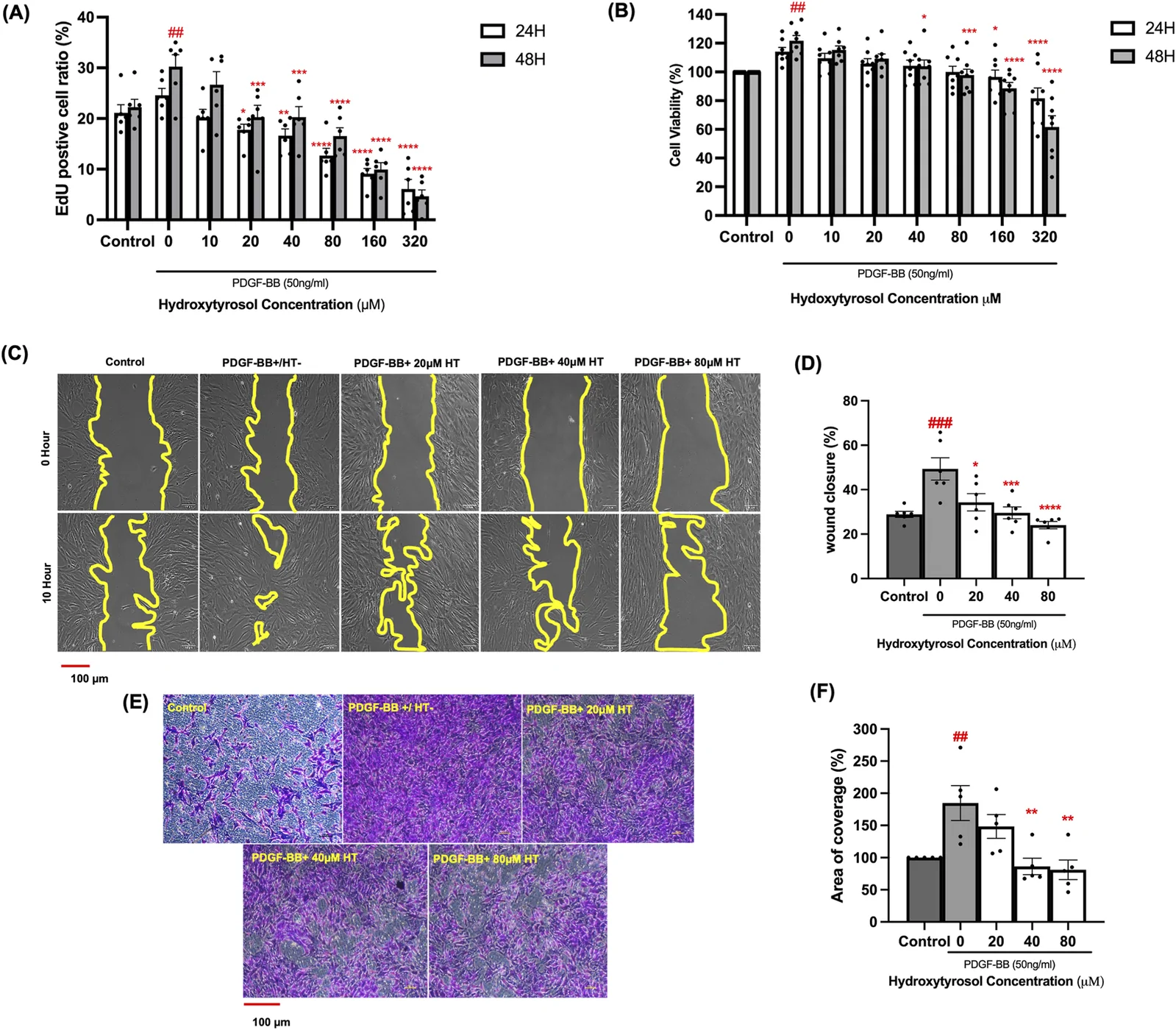
**(A,B)** Effect of hydroxytyrosol (HT) on PDGF-BB–induced SMC proliferation and viability. PDGF-BB (50 ng/mL)–stimulated SMCs were cultured for 24 and 48 h with HT alone. **(A)** Cell proliferation was assessed using the EdU assay. EdU-positive cells were visualized by fluorescence microscopy, and quantitative analysis was performed using ImageJ software (N = 6; n = 3). **(B)** Cell metabolic activity/viability was assessed using the MTT assay. Absorbance values were converted to percentage metabolic activity/viability. Cell viability and proliferation were normalized to the respective control. Data are presented as mean ± SEM, ##p < 0.01 versus 24/48 h control; *p < 0.05, **p < 0.01, ***p < 0.001, ****p < 0.0001 versus respective 24/48 h PDGF-BB–induced group (two-way ANOVA, Dunnett’s multiple comparisons test). **(C,D)** Effect of HT on PDGF-BB–induced SMC migration. Untreated cells served as control. **(C)** Representative images at 0 and 10 h (×100 magnification; scale bar = 100 μm). **(D)** Quantitative analysis of wound width normalized to the respective control (N = 5; n = 3). **(E–F)** Effect of HT on PDGF-BB–induced SMC transwell migration. Untreated cells served as control. **(E)** Representative images of migrated cells (100× magnification; scale bar = 100 μm; N = 3; n = 3). **(F)** Quantitative analysis of migrated cells. Data are presented as mean ± SEM, ##p < 0.01, ###p < 0.001 versus control; *p < 0.05, **p < 0.01, ***p < 0.001, ****p < 0.0001 versus respective PDGF‐BB–induced group (one-way ANOVA, Dunnett's multiple comparisons test). N = biological replicates; n = technical replicates. The plotted values represent the average of technical replicates (n = 3) obtained from each donor (N = 5–8), as specified.

### 3.2 Effect of HT in EC metabolic activity/viability, proliferation, migration, and transwell migration

Cell proliferation analyses (Figure 3A) showed that HT concentrations ranging from 6.25 to 50 μM did not significantly affect ECs. For example, at 24 h, the proliferation rate of control ECs was 56.23% ± 6.6% and remained at 49.41% ± 6.06% at 50 μM HT treatment (p = 0.9321). Similar results were obtained after 48 h of 50 μM HT treatment on ECs proliferation rate (55.9% ± 5.45% vs. 48.8% ± 6.15%, p = 0.9233). Increasing HT concentrations (≥100 μM) significantly reduced EC proliferation compared with the untreated control, with proliferation rates decreasing by 49.69% and 55.22% after 24 h and 48 h, respectively. Hydroxytyrosol (HT) did not significantly affect EC metabolic activity/viability at concentrations up to 100 μM after either 24 h or 48 h of treatment (Figure 3B). At 48 h, treatment with 200 μM HT significantly reduced EC metabolic activity/viability by 21.25% compared with the untreated control, resulting in a viability of 78.75% ± 8.50% (p = 0.0004). For the wound closure assay, 6.25–50 μM doses of HT were used. Images were taken at 0 and 24 h, as shown in Figure 3C. No significant difference in wound closure was observed compared to the control at these doses (Figure 3D). In addition to this, the transwell migration test demonstrated no significant changes in HT-treated samples compared to the control (Figures 3E,F).

**FIGURE 3:**
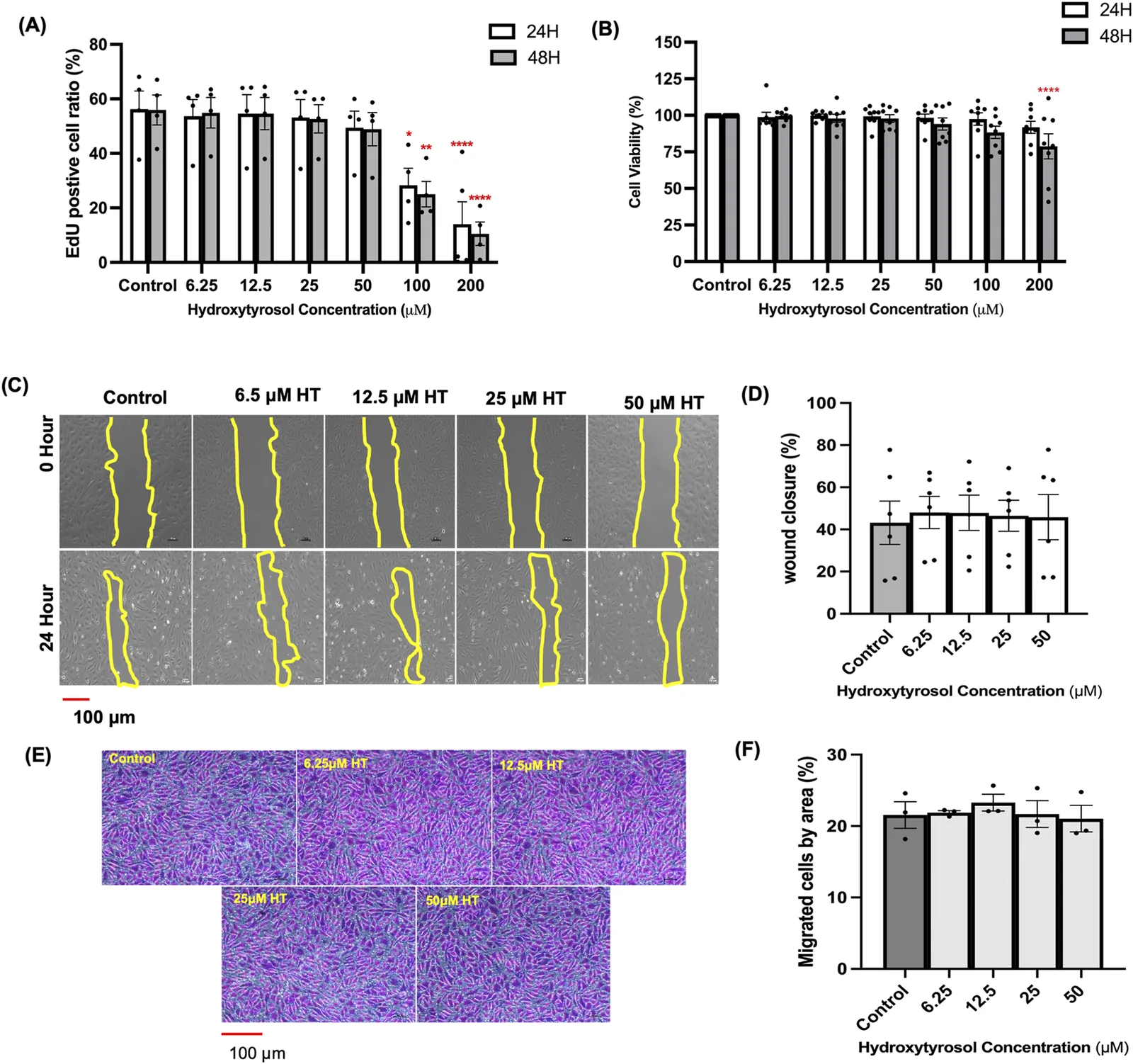
**(A,B)** Effect of hydroxytyrosol (HT) on EC proliferation and viability. Cells were treated with HT (6.25, 12.5, 25, 50, 100, 200 μM) for 24 and 48 h. Untreated cells served as control. **(A)** Cell proliferation was assessed using the EdU assay. EdU-positive cells were visualized by fluorescence microscopy, and quantitative analysis was performed using ImageJ software (N = 5; n = 3). **(B)** Cell viability was assessed using the MTT assay. Absorbance values were converted to percentage cell viability (N = 8; n = 3). Cell proliferation and viability were normalized to the respective control. Data are presented as mean ± SEM, *p < 0.05, **p < 0.01, ****p < 0.0001 versus 24/48 h control (two‐way ANOVA, Dunnett's multiple comparisons test). **(C,D)** Effect of HT on EC migration. Untreated cells served as control. **(C)** Representative images of control and treated groups (scale bar = 100 μm). **(D)** Quantitative analysis of migrated area, normalized to the respective control (N = 6; n = 3). **(E,F)** Effect of HT on EC transwell migration. Untreated cells served as control. **(E)** Representative images of crystal violet–stained migrated ECs (scale bar = 100 μm; N = 3; n = 3). **(F)** Quantitative analysis of migrated cells measured using ImageJ software. Data are presented as mean ± SEM, one-way ANOVA, Dunnett’s multiple comparisons test. N = biological replicates; n = technical replicates. The plotted values represent the average of technical replicates (n = 3) obtained from each donor (N = 5–8), as specified.

### 3.3 Differential effects of HT on Akt and Nrf2/HO-1 signalling in ECs and SMCs

In SMCs, PDGF-BB significantly increased Akt phosphorylation (Ser473), as reflected by an increased pAKT/tAKT ratio of 1.97 ± 0.25 compared with the control group (p = 0.0044). HT treatment was associated with a 36.6% reduction in Akt phosphorylation, resulting in a pAKT/tAKT ratio of 1.25 ± 0.17 (p = 0.0283 vs. PDGF-BB) (Figures 4A,B). The expression of the antioxidant-related proteins Nrf2 and HO-1 was also evaluated (Figures 4A,C,D). HT-treated SMCs exhibited significantly higher Nrf2 expression than both the control and PDGF-BB groups, reaching 3.7 ± 1.0 compared with 1.2 ± 0.45 in the PDGF-BB group (p = 0.0369). HO-1 expression showed a similar trend, increasing from 3.6 ± 1.8 in the PDGF-BB group to 4.5 ± 2.0 following HT treatment; however, these differences were not statistically significant (p > 0.05). In ECs (Figures 4E–H), treatment with 50 μM HT significantly increased the pAKT/tAKT ratio after 1 h, reaching 1.95 ± 0.21 (p = 0.0004) (Figures 4E,F). Similarly, Nrf2 expression was significantly increased after 1 h of HT treatment (2.91 ± 0.55, p = 0.0175) (Figures 4E,G). HO-1 expression increased in a time-dependent manner, reaching 1.88 ± 0.31 at 1 h (p = 0.7182) and 8.06 ± 1.53 after 48 h (p = 0.0001) (Figure 4E).

**FIGURE 4:**
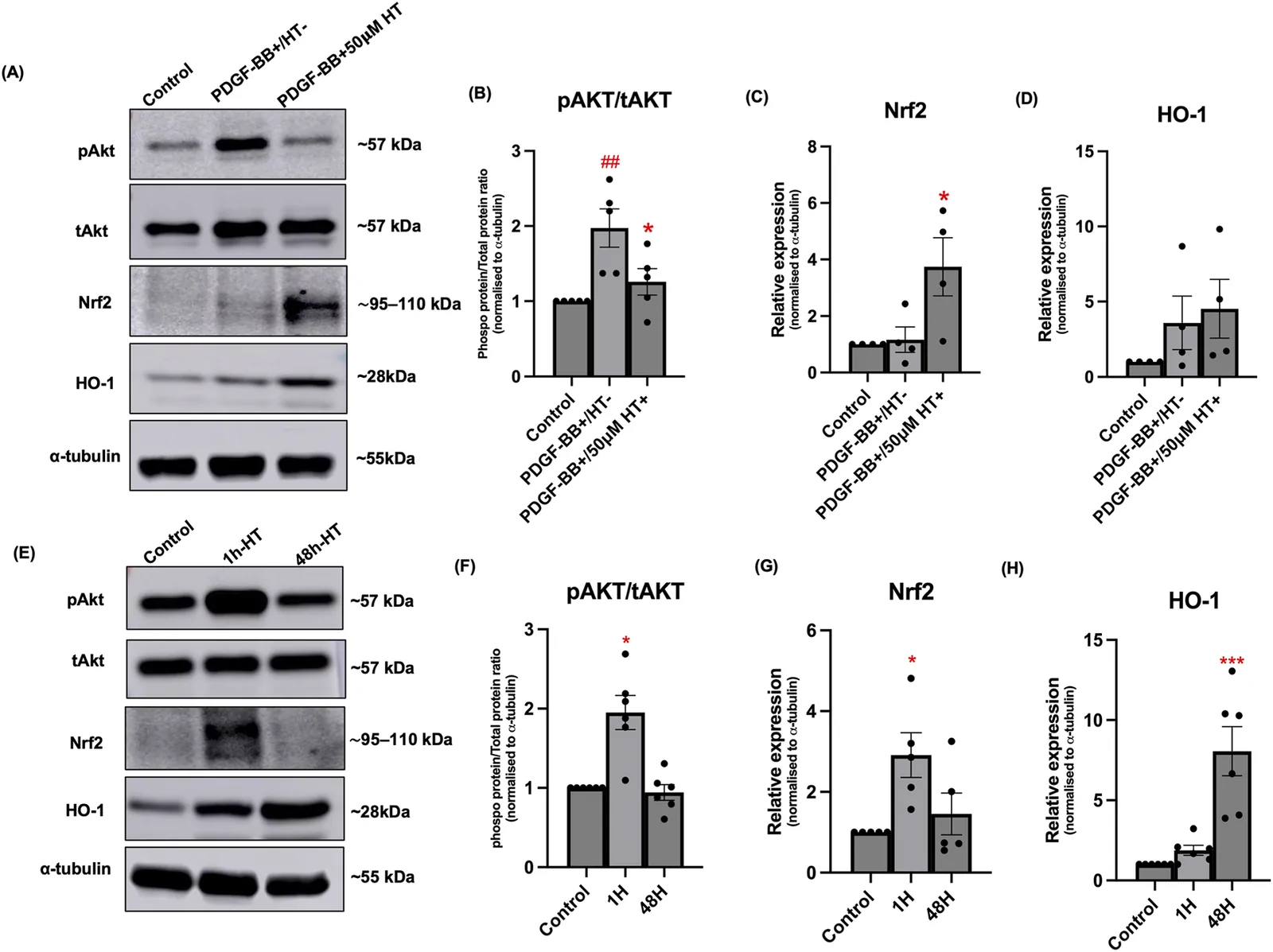
Effect of hydroxytyrosol (HT) on AKT and Nrf2/HO-1 signaling pathways in SMCs and ECs. **(A–D)** Effect of HT on pAkt(Ser473)/total Akt, Nrf2, and HO-1 expression in PDGF-BB–stimulated SMCs. SMCs were treated with PDGF-BB with or without HT (50 μM) for 48 h. **(A)** Representative Western blot images for control, PDGF-BB, and PDGF-BB + HT groups. **(B–D)** Quantitative analysis of pAkt (Ser473)/total Akt, Nrf2, and HO-1 expression. Data are presented as mean ± SEM, ##p < 0.01 versus control; *p < 0.05 versus PDGF-BB+/HT− group (one-way ANOVA, Dunnett’s multiple comparisons test). HO-1: N = 4; n = 3; Nrf2: N = 4; n = 3. **(E–H)** Effect of HT on pAkt(Ser473)/total Akt, Nrf2, and HO-1 expression in ECs. ECs were treated with HT (50 μM) for 1 h or 48 h, as indicated. Untreated cells served as control. **(E)** Representative Western blot images for each experimental condition. **(F–H)** Quantitative analysis of pAkt(Ser473)/total Akt, Nrf2, and HO-1 expression. α-tubulin was used as an internal loading control for normalization. Data are expressed relative to control. Data are presented as mean ± SEM, *p < 0.05, ***p < 0.001 versus control (one-way ANOVA, Dunnett’s multiple comparisons test). pAkt/tAkt: N = 6; n = 3; HO-1: N = 6; n = 3; Nrf2: N = 5; n = 3. N = biological replicates; n = technical replicates.

### 3.4 Hydroxytyrosol effect in *ex vivo* hSV ring culture model (hSVR)

We next evaluated the effect of 50 μM HT on spontaneous intimal thickening in *ex vivo* human saphenous vein rings (hSVRs). Intimal morphology and thickness were assessed over 21 days of culture (Figures 5A,B). Although baseline vein segments exhibited some pre-existing intimal thickening consistent with donor vascular disease, untreated rings showed a further significant increase in intimal thickness over the 21-day culture period (96.10 ± 12.83 μm at day 0–155.02 ± 18.79 μm at day 21, p = 0.0045), demonstrating that the model captures active progression of intimal hyperplasia beyond baseline. HT treatment significantly attenuated this progression. Direct comparison between untreated and HT-treated rings revealed no significant differences at days 0, 5, 10 or 14. However, by day 21, HT-treated rings exhibited significantly lower intimal thickness than untreated controls (85.01 ± 3.52 μm vs. 155.02 ± 18.79 μm, p = 0.0014), representing an approximately 45.2% reduction in intimal thickness (Figure 5B). Media thickness was similarly reduced in HT-treated cultures compared with control at later timepoints during the 21-day culture period (Supplementary Figure S2). The intima/media ratio did not differ significantly between groups or across timepoints (Supplementary Figure S3), indicating that HT reduced overall vessel wall thickness (both intima and media) proportionally, without altering the relative distribution between the two compartments. Cell proliferation within the intimal region was assessed by EdU incorporation (Figures 5C,D). Untreated rings exhibited a significantly higher percentage of EdU-positive cells at day 21 compared with day 5 (61.33% ± 6.96% vs. 23.18% ± 6.09%, p = 0.0007). HT significantly reduced EdU-positive cells at day 21 compared with untreated controls (28.66% ± 4.66% vs. 61.33% ± 6.96%, p = 0.0100). The effect of HT on re-endothelialisation was further examined by seeding cultured ECs onto hSVRs (Figure 5E). CD31-positive cells were detected in EC-seeded rings with and without HT treatment but were predominantly localized to the adventitial region, with minimal endothelial coverage along the luminal surface. Therefore, the effect of HT on re-endothelialisation could not be conclusively determined in this model.

**FIGURE 5:**
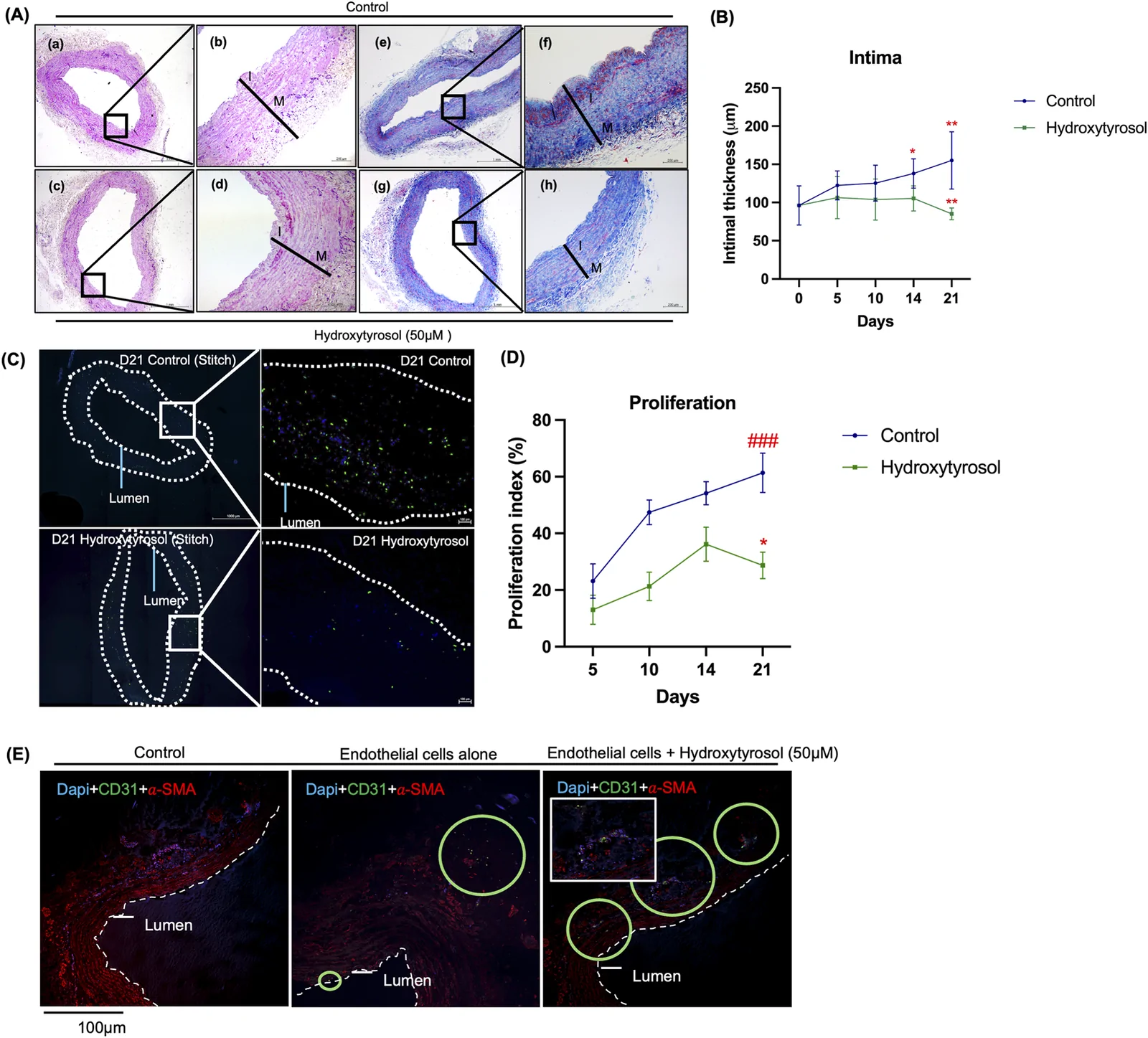
Effect of hydroxytyrosol (HT) on intimal hyperplasia in *ex vivo* human saphenous vein rings (hSVRs). Vein rings were cultured for up to 21 days with or without HT (50 μM). **(A)** Representative H&E and Masson’s trichrome-stained sections of untreated and HT-treated hSVRs at day 21. Higher-magnification images are shown in adjacent panels. I = intima; M = media. **(B)** Quantitative analysis of intimal thickness over the culture period. Data are presented as mean ± SEM (N = 5; n = 3). On the control line, *p < 0.05 and **p < 0.01 versus day 0 control; on the hydroxytyrosol line, **p < 0.01 versus corresponding day 21 control. **(C)** Representative EdU staining images showing proliferating cells (green) and nuclei (blue) within the neointimal region. **(D)** Quantitative analysis of EdU-positive cells. Data are presented as mean ± SEM (N = 5; n = 3). ###p < 0.001 versus day 5 control; *p < 0.05 versus corresponding day 21 control. **(E)** Assessment of re-endothelialisation in hSVRs stained with CD31 (green), α-SMA (red), and DAPI (blue). Green circles indicate CD31-positive endothelial cells. Scale bar = 100 μm. N = biological replicates; n = technical replicates.

### 3.5 Hydroxytyrosol increased HO-1 expression and reduced Akt phosphorylation and vimentin expression in hSVRs

On day 0, the expression of pAKT/tAKT was 1.10 ± 0.51 (Figure 6B). The ratio progressively increased from day 5 and reached statistical significance on days 14 and 21 (3.58 ± 0.41 and 3.34 ± 0.32, respectively) compared to day 0. On day 5 the pAkt (Ser473)/total Akt ratio was already diminished in HT-treated hSVRs compared to day 5 of untreated hSVRs and was overall lowered compared to untreated hSVRs along the whole-time course. HT treatment significantly reduced the pAKT(Ser473)/total AKT ratio compared with the untreated day 10 control (0.80 ± 0.23 vs. 2.72 ± 0.24) for control day 10, p = 0.045) (Figures 6A,B). HO-1 expression was evaluated in hSVRs throughout culture (Figures 6A,C). In untreated control rings, HO-1 expression increased significantly from 6,387 ± 3,829 at day 0–19,590 ± 1,583 at day 10 (p = 0.0062), before declining to 12,065 ± 2016 at day 14 and returning to baseline by day 21 (5,505 ± 915). Although HO-1 expression was higher in HT-treated rings than untreated controls at all time points, statistically significant differences were observed only at days 14 and 21. HO-1 expression reached 22,112 ± 2,456 in HT-treated hSVRs at day 14 compared with 12,065 ± 2016 in untreated controls (p = 0.0352), and remained elevated at day 21 (15,633 ± 2,368 vs. 5,505 ± 915, p = 0.0335). No significant differences were detected between HT-treated and untreated groups at days 5 or 10. Vimentin expression (Figure 6D) was subsequently evaluated in the *ex vivo* IH model with and without HT treatment. Vimentin expression increased over time in untreated hSVRs, suggesting phenotypic modulation and cellular remodeling associated with intimal hyperplasia development. In contrast, HT treatment was associated with lower vimentin expression compared with untreated controls. No significant differences in vimentin expression were observed in PDGF-BB-induced SMCs *in vitro* (data not shown); therefore, vimentin expression was further evaluated in the *ex vivo* model. At day 0, vimentin expression in control hSVRs was 1723 ± 347. Expression increased significantly by day 14 (15,106 ± 1,018) and remained elevated at day 21 (10,545 ± 1845), suggesting a suggesting cellular remodeling associated with intimal hyperplasia development. HT treatment markedly attenuated this increase, reducing vimentin expression to 2,868 ± 591 at day 14 and 1,494 ± 591 at day 21.

**FIGURE 6:**
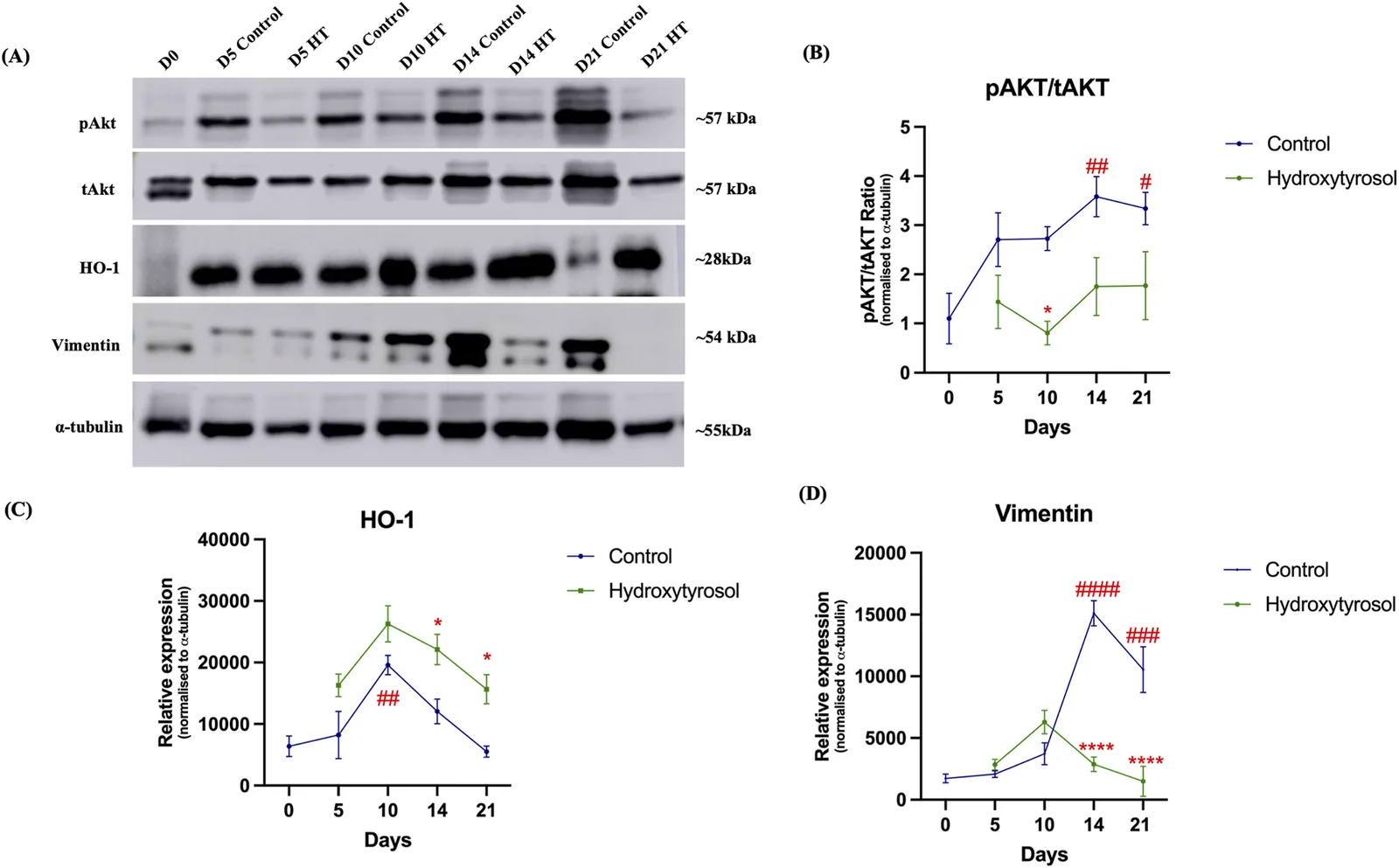
Effect of hydroxytyrosol (HT) on pAKT, tAKT, HO-1, and vimentin expression in *ex vivo* saphenous vein rings (SVRs). SVRs were cultured with or without HT (50 μM) for up to 21 days. On days 5, 10, 14, and 21, rings were lysed and protein expression was analyzed by Western blotting. Day 0 (D0) and media-only treated SVRs served as controls. **(A)** Representative western blot images showing pAKT, tAKT, HO-1, vimentin, and α-tubulin expression across timepoints and treatment conditions. **(B–D)** Quantitative analysis of protein expression. **(B)** pAKT/tAKT ratio in the intimal hyperplasia (IH) model. **(C)** HO-1 expression in the IH model. **(D)** Vimentin expression in the IH model. Band intensities were quantified using ImageJ software and normalized to α-tubulin. Data are presented as mean ± SEM (one-way and two-way ANOVA, Dunnett’s multiple comparisons tests). #p < 0.05, ##p < 0.01, ###p < 0.001, ####p < 0.0001 versus D0 group; *p < 0.01, ****p < 0.0001 versus respective day control group. N = 3 biological replicates; n = 3 technical replicate.

## 4 Discussion

Intimal Hyperplasia (IH) arises from a complex interplay of dysfunctional ECs and SMCs following vascular injury. This dysfunction leads to uncontrolled cellular proliferation, creating a self-perpetuating cycle of pathological changes (Melnik et al., 2022). Functional ECs are crucial in mitigating IH by suppressing key pathological events, including inflammation and thrombus formation (Ladak et al., 2022). However, the lack of studies focusing on endothelial toxicity has hindered the development of effective pharmacological therapies for IH. Consequently, the development of therapeutic strategies that suppress pathological SMC responses while maintaining endothelial integrity remains an important objective in the prevention of restenosis and vein graft failure.

Our study demonstrated that HT effectively inhibited PDGF-BB-induced activation of the proliferative phenotypes in the SMC without compromising cell viability at concentrations below 40 μM HT. Additionally, HT significantly suppressed PDGF-BB-induced SMC migration in a wound healing assay, demonstrating a dose-dependent inhibition across all tested concentrations (20–80 μM). HT also reduced the transwell migration of PDGF-BB-stimulated SMCs. These findings align with previous studies reporting that HT counteracted PDGF-induced cell proliferation and chemotaxis of aortic SMCs (Zrelli et al., 2011a). Other reports have similarly demonstrated that the olive-derivative oleuropein and HT significantly inhibited SMC proliferation at a concentration of 100 μM, whereas lower doses had no effect (Abe et al., 2012). Furthermore, several naturally derived compounds have been shown to counteract the potent mitogenic effects of PDGF-BB on SMCs, highlighting their potential as therapeutic agents or co-adjuvants for preventing restenosis or atherosclerosis (Hsuan et al., 2020; Fan et al., 2021). Most previous studies have focused on SMC antimitogenic compounds. HT has been reported to exhibit a favorable safety profile and is classified as a Generally Recognized as Safe (GRAS) substance by the FDA. Nevertheless, the concentration-dependent effects observed in the present study highlight the importance of defining an appropriate therapeutic range for vascular applications.

Our findings provide a key advancement by demonstrating that HT not only inhibits SMCs hyperproliferation but also preserves overall endothelial cell function (including viability, proliferation, and migration), while maintaining SMCs in a non-activated phenotype. Moreover, HT is not associated with adverse effects, mutagenicity, or genotoxicity (Bertelli et al., 2020; Pastor et al., 2021). Its designation as a Generally Recognized as Safe (GRAS) substance by the FDA (Turck et al., 2017) further supports its potential for rapid translation. Moreover, our overall findings are consistent with previous studies investigating the effect of HT on the EC function (Zrelli et al., 2011b; Cheng et al., 2017; Abate et al., 2020). These studies reported that HT, at concentrations ranging from 50 to 100 μM, significantly increased the proliferation of porcine ECs (Zrelli et al., 2011b; Zrelli et al., 2011c). Similarly, Cheng et al. found that HT maintained EC proliferation at concentrations between 10 and 30 μM, though a significant reduction was observed at 50 μM (Cheng et al., 2017). Additionally, high-dose HT was shown to sustain the cell viability of human umbilical vein endothelial cells (HUVEC) at concentrations up to 160 μM (Abate et al., 2020). Previous *ex vivo* models reported loss of tissue viability after 14 days, with SMCs exhibiting diminished contractility and eventual necrosis in response to vasoactive stimuli. Similarly (Clowes and Clowes, 1985), reported that baseline by day 28, connective tissue and intima volume returned to baseline (Clowes and Clowes, 1985). While similar *ex vivo* saphenous vein ring models developed by Schachner et al. (2007) and Wong et al. (2016), were only maintained for 14 days (Schachner et al., 2007; Wong et al., 2016). In contrast, our study successfully extended the culture duration to 21 days without significant cell loss or intimal regression. This improvement likely stems from our use of an endothelial growth medium-2 (EGM-2, Promocell) supplemented with growth factors such as fetal calf serum (FCS), epidermal growth factor (EGF), basic fibroblast growth factor (bFGF), ascorbic acid, heparin, and hydrocortisone rather than the basic RPMI medium employed in prior studies.

Importantly, we demonstrated that HT significantly suppressed intimal thickening at day 21. Both untreated and HT-treated rings showed an increase in intimal thickness over the 21-day culture period, but the untreated increase reached significance earlier (by day 14) and continued to rise, whereas the HT-treated increase remained non-significant throughout and only diverged clearly from control by day 21. This time course is consistent with the chronic nature of intimal hyperplasia, where early neointimal changes reflect SMC migration into the intima, followed by a slower phase of sustained SMC proliferation and progressive extracellular matrix accumulation (Melnik et al., 2022) that drives most of the measurable thickening. The delayed divergence between groups suggests HT acts primarily by blunting this later proliferative/matrix-accumulation phase rather than preventing the initial migratory response, which would explain why a treatment effect on thickness was not detectable until day 21 despite HT’s effects on SMC proliferation being evident much earlier in the 2D culture assays. We coculture endothelial cells (ECs) with human saphenous vein (hSV) rings to assess HT’s impact on endothelial recovery. Manual seeding was necessary due to the preexisting endothelial denudation, consistent with reports linking basal intimal thickness (>120 μm) to endothelial dysfunction (Li et al., 2013). All our samples exhibited preexisting intimal hyperplasia, likely explaining the lack of luminal endothelial coverage, a finding supported by our prior work and studies noting impaired endothelial function in older patients’ vein grafts (Haron et al., 2024; Saemann et al., 2023). While EC-seeded rings (with or without HT) showed minimal EC presence in the adventitia, possibly from neovascularization, no luminal repopulation was observed. Further validation using 3D models with dynamic flow or animal studies is warranted to better assess HT’s role in endothelialization under physiological conditions. Mechanistically, the PI3K/AKT plays a central role in IH development by promoting SMCs’ proliferation, migration, and phenotypic switching upon vascular injury (Melnik et al., 2022; Shan et al., 2022).

Our systematic literature search revealed that HT potentially modulates the PI3K/AKT/mTOR pathway, consistent with our findings and suggesting a role for HT in inhibiting IH progression (Vijakumaran et al., 2021). Antiproliferative-coated drug-eluting stents (Sirolimus-DES) also target this pathway to induce cell cycle arrest. Other natural compounds, including Andrographolide (Han et al., 2023), Astragalus, Berberine (BBR) (Zhang et al., 2021), Angelica (Yan et al., 2018), and Hesperetin (Wei et al., 2016), have been reported to inhibit neointimal formation by to deactivating PI3K/AKT signaling. Beyond PI3K/AKT inhibition, phytochemicals such as resveratrol, lycopene, or luteolin have demonstrated PI3K/Akt-mediated activation of Nrf2/HO-1 signaling in pathological conditions involving oxidative stress, apoptosis, and inflammation, key processes in atherosclerosis (Saha et al., 2020; Xu et al., 2022). Our study showed that HT treatment was associated with reduced Akt signalling in PDGF-BB-stimulated SMCs, in which Akt activation was significantly elevated compared with untreated controls. Consistent with our findings, PDGF-induced Akt signalling has been shown to drive Cyclin D1 expression and promote cell-cycle progression in vascular smooth muscle cells, while inhibition of Akt reduces proliferation and causes G1/S arrest by modulating cell-cycle regulators such as p21^Cip1, supporting the role of Akt as a critical upstream regulator of SMC proliferation (Stabile et al., 2003; Martin-Garrido et al., 2013). Previous studies have demonstrated that magnobovatol inhibits PI3K/Akt signaling in vascular smooth muscle cells, resulting in decreased MMP-2/9 expression and impaired migratory capacity, further supporting the concept that Akt serves as a key upstream regulator of both proliferative and migratory pathways in vascular remodelling (Kang et al., 2016).

Additionally, HT upregulated Nrf2 and HO-1 in these cells. The Nrf2/HO-1 pathway is well known for its antioxidant, anti-inflammation, and cytoprotective properties (Hassanein et al., 2023). Notably, Nrf2 depletion enhances SMC migration *in vitro*, and Nrf2-deficient mice exhibited enhanced IH (Ashino et al., 2013). Other compounds, such as procyanidins, have shown to counteract oxidative stress-induced cytotoxicity in SMCs by activating Nrf2 (Zhang et al., 2021) while the antidiabetic drug canagliflozin suppressed SMC proliferation and migration via Nrf2/HO-1 activation (Behnammanesh et al., 2020). Similarly, hydrogen sulfide has been found to reduce IH by activation of the Nrf2 signaling (Ling et al., 2021). Our findings suggest that HT’s suppression of PDGF-BB-induced migration is likely associated with Nrf2/HO-1 activation. Apart from its antioxidant role, HO-1 also directly regulates intimal hyperplasia. It inhibits neointimal hyperplasia formation in balloon-injured rats by enhancing nitric oxide production (Cerrito et al., 2011) and suppressing IH in an arteriovenous fistula (AVF) dysfunction via HO-1/PPAR-γ pathway (Xie et al., 2022). Additionally, HO-1 has been recognized as an antiproliferative factor in cancer studies (Romeo et al., 2021; Chien et al., 2022), further underscoring its potential in controlling IH.

Consistent with its effect on SMCs, HT also upregulated Nrf2 and HO-1 in ECs. We observed a significant increase in Nrf2 expression within 1 hour of treatment, whereas HO-1 levels peaked at 48 h. This delayed HO-1 response may be explained by the short half-life of Nrf2 (20 min) which, upon stabilization, can extend up to 200 min (Canning et al., 2015; Loboda et al., 2016). Since HO-1 is downstream of Nrf2, its expression remains detectable even after Nrf2 levels return to baseline. Nrf2 is known to enhance EC function by regulating oxidative stress, exerting anti-inflammatory, antithrombotic, and antiapoptotic effects (Zhang et al., 2021). Similarly, HT has been shown to activate Nrf2/HO-1 in vascular endothelial wound healing (Zrelli et al., 2015) and protect against oxidative stress by activating this pathway (Zrelli et al., 2011b). Other polyphenols, such as resveratrol, have been reported to activate the Nrf2/HO-1 pathway through the PI3K/AKT pathway, protecting cells from oxidative stress and apoptosis (Xu et al., 2022). Our findings suggest that HT exert cytoprotective effects in ECs are associated with Nrf2/HO-1 modulation, contributing to vascular homeostasis and reinforcing its potential as a therapeutic agent for IH (Pan et al., 2020). Additionally, HO-1 expression was significantly higher in the HT-treated IH model, consistent with *in vitro* findings. HO-1 has been shown to protect against IH in arteriovenous fistula dysfunction by balancing oxidative stress (Xie et al., 2022). However, despite multiple attempts, Nrf2 was undetectable, likely due to its short half-life (∼20 min) and rapid degradation via proteasomes under basal conditions (Stewart et al., 2003). Upon oxidative or electrophilic stimulation, Nrf2 is transiently stabilized and rapidly translocates to the nucleus, where it binds to antioxidant response elements (AREs) to initiate transcription of cytoprotective genes, after which it is again targeted for proteasomal degradation (Kensler et al., 2007; Ma, 2013). This tightly regulated and dynamic activation cycle may limit detection of total Nrf2 protein in fixed *ex vivo* tissues. In contrast, heme oxygenase-1 (HO-1), a canonical downstream transcriptional target of Nrf2, exhibits more sustained expression and is commonly used as a functional surrogate marker of Nrf2 pathway activation, as HMOX1 is widely recognized among the core NRF2-regulated antioxidant genes across diverse models (Morgenstern et al., 2024). Further, reduced vimentin expression in HT-treated hSVRs was interpreted as a marker of attenuated mesenchymal activation and tissue remodeling; however, vimentin is a non-specific mesenchymal marker also expressed by fibroblasts, adventitial cells, and activated ECs, and its measurement in whole vein-ring lysates reflects contributions from multiple cell types rather than SMCs exclusively. This finding should therefore be interpreted as supportive, rather than definitive, evidence of SMC phenotypic modulation.

## 5 Limitation

A key limitation of our study is the inconsistent endothelial coverage observed in the hSV samples. While endothelial cells were successfully isolated from a small subset of samples, the majority did not yield viable endothelium, likely due to surgical manipulation (e.g., distension and valve reversal) during graft preparation. While this may reflect real-world clinical scenarios where damaged venous segments are repurposed for arterial bypass, it limits our ability to assess HT’s effects on reendothelialization. Additionally, since SVs are harvested from patients with underlying vascular disease, our model may better reflect real-world pathological conditions requiring anti-restenosis therapy. Nevertheless, our findings demonstrate HT’s protective potential in damaged vascular tissues. Future studies should prioritize intact saphenous veins models cultured under physiological perfusion system to better mimic arterial hemodynamics and elucidate intimal hyperplasia progression under native-like pressure conditions. Additionally, HT’s reendothelialization efficacy warrants evaluation in balloon-injury models, which offer more reliable endothelial repair assessments than our current *ex vivo* setup. Additionally, we did not assess hydroxytyrosol stability in culture medium or exclude extracellular hydrogen peroxide generation, which is a recognised property of catechols including hydroxytyrosol in bicarbonate-buffered cell culture. Future studies incorporating catalase co-treatment or direct measurement of medium H2O2 and hydroxytyrosol stability are needed to clarify the relative contribution of direct hydroxytyrosol signalling versus extracellular H2O2 to the effects reported here. Dietary HT reaches plasma only in the nanomolar range due to extensive first-pass sulfation/glucuronidation, so the concentrations used here were not intended to model systemic oral exposure. Instead, 50 µM was selected as the lowest dose effectively inhibiting SMC proliferation/migration while preserving endothelial function, consistent with the higher concentrations typically needed *in vitro*/*ex vivo* due to limited uptake, culture-media oxidation, and absent first-pass metabolism. These findings may be more relevant to localized delivery approaches (e.g., graft-soak or perivascular delivery), where tissue concentrations can exceed systemic levels, future work will test the major HT sulfate/glucuronide metabolites at physiologically relevant concentrations.A further limitation is that SMCs and ECs were derived from non-matched donors; consequently, the apparent cell-type selectivity of the therapeutic window cannot be fully disentangled from inter-patient variability, and this should be confirmed in future studies using donor-matched cell pairs. A limitation of this study is that cross-sectional intimal area and covariate associations (age, sex, diabetes, smoking, statin/antiplatelet use, renal function) with baseline intima or hydroxytyrosol response were not assessed, as the cohort was not powered for such analyses.

## 6 Conclusion

In conclusion, hydroxytyrosol (HT) inhibited PDGF-BB-induced smooth muscle cell proliferation and migration, without producing a statistically significant reduction in endothelial cell viability, proliferation, or migration, under the experimental conditions examined. These cellular responses were accompanied by reduced Akt activation in SMCs, increased Nrf2/HO-1 signalling in ECs, and modulation of Akt, HO-1, and vimentin expression in the *ex vivo* human saphenous vein ring model. Furthermore, HT attenuated the progression of pre-existing intimal thickening and reduced proliferative activity within the neointima during *ex vivo* culture. Although the effect of HT on re-endothelialisation could not be conclusively determined, these findings support the potential of HT as a naturally derived compound for limiting vascular remodelling associated with vein graft intimal hyperplasia. Further studies using perfused vascular models and *in vivo* systems are required to clarify the underlying mechanisms and evaluate its translational potential.

## Data Availability

The raw data supporting the conclusions of this article will be made available by the authors, without undue reservation, on request from the corresponding author.
