# Supplementary material for: Hydroxytyrosol attenuates intimal hyperplasia in in vitro and ex vivo human saphenous vein models: association with Akt and Nrf2/HO-1 signalling
Source: Front Pharmacol. 2026 Oct 28;17:1962884. doi: 10.3389/fphar.2026.1962884 (PMC13751944; doi:10.3389/fphar.2026.1962884)
Supplement: Supplementary file 1 [file DataSheet1.pdf]

Supplementary Table S1

Demographic and clinical characteristics of patients undergoing coronary artery bypass grafting (CABG) from whom surplus human saphenous vein samples were obtained for ex vivo studies.

**Characteristic**	**Value (n = 35)**
Mean age (years)	59 ± 9.2
Male, n (%)	28 (80%)
Diabetes mellitus type II, n (%)	13 (37%)
Hypertension, n (%)	28 (80%)
Hyperlipidaemia, n (%)	22 (63%)
Obesity, n (%)	2 (6%)
History of smoking, n (%)	14 (40%)
Chronic kidney disease, n (%)	4 (11%)
Fatty liver disease, n (%)	2 (6%)
Transient ischaemic attack (TIA)/mini stroke, n (%)	3 (9%)

Data are presented as mean ± standard deviation or number (%). Patient characteristics were obtained from individuals undergoing CABG surgery at Hospital Canselor Tuanku Muhriz, Universiti Kebangsaan Malaysia, from whom surplus saphenous vein graft segments were collected for ex vivo experimentation.

Supplementary Table S2. Antibody Details

Primary Antibodies

Primary Antibodies	Host Species	Application / Dilution	Catalogue Number	Manufacturer
α-Smooth Actin	Mouse	ICC: 1/250; IHC: 1/250; WB: 1:1000	ab7817	Abcam
CD31	Mouse	ICC: 1/150	ab9498	Abcam
Vimentin	Rabbit	WB: 1/2000	5741S	Cell Signaling Technology
Phospho-Akt (Ser473)	Rabbit	WB: 1/1000	4060S	Cell Signaling Technology
Pan-Akt	Rabbit	WB: 1/1000	4685S	Cell Signaling Technology
HO-1	Rabbit	WB: 1/1000	43966S	Cell Signaling Technology
NRF2	Rabbit	WB: 1/1000	33649S	Cell Signaling Technology
α-Tubulin	Rabbit	WB: 1/1000	2125S	Cell Signaling Technology

Secondary Antibodies

Secondary Antibody	Target Species	Application / Dilution	Catalogue Number	Manufacturer
Goat Anti-Rabbit IgG (Alexa Fluor 488)	Rabbit	IF: 1:400	ab150077	Abcam
Goat Anti-Mouse IgG (Alexa Fluor 594)	Mouse	IF: 1:400	ab150116	Abcam
Goat Anti-Rabbit IgG (HRP-linked)	Rabbit	WB: 1:10000	7074P2	Cell Signaling Technology
Goat Anti-Mouse IgG (HRP)	Mouse	WB: 1:10000	7075P2	Cell Signaling Technology

Supplementary S1

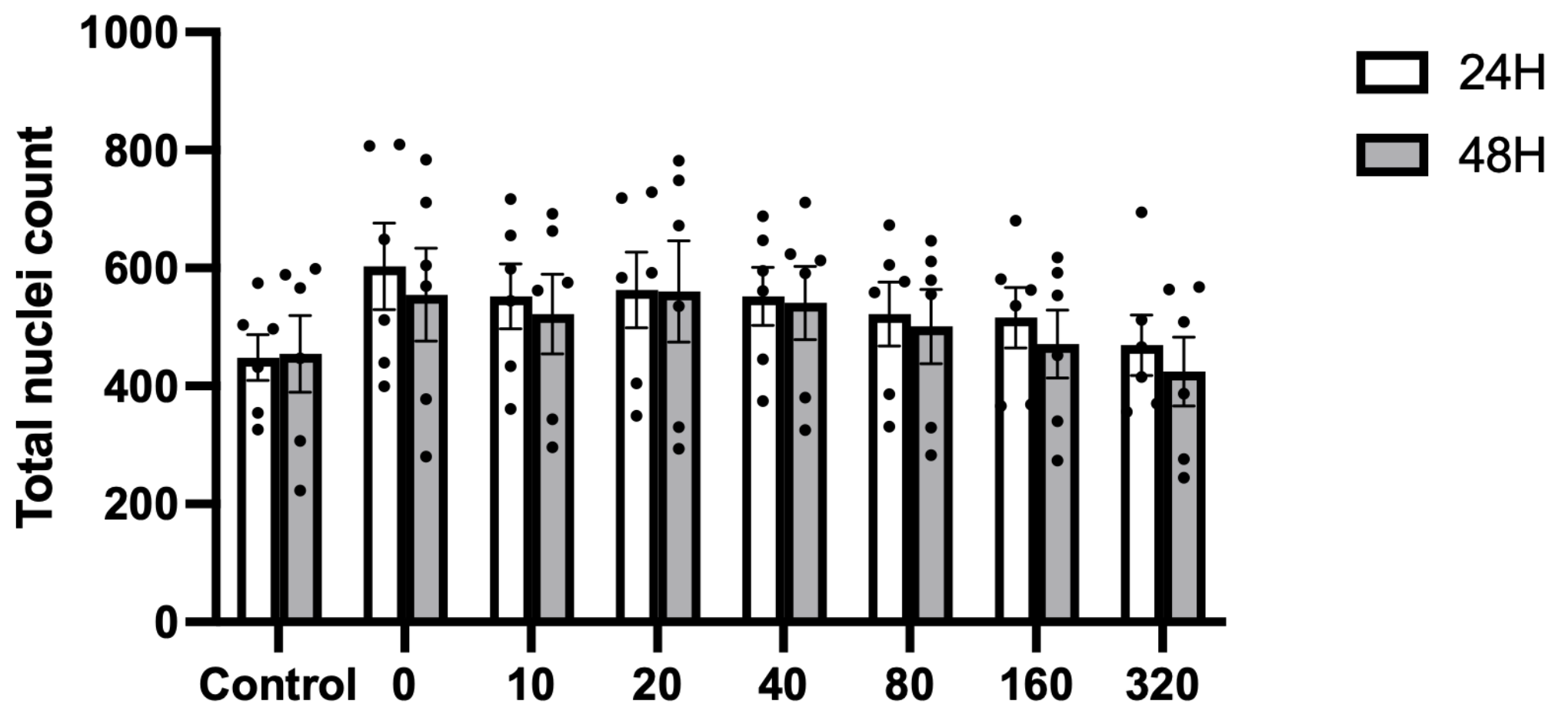

Supplementary Figure S1. Hydroxytyrosol does not reduce total nuclei of smooth muscle cells number at any tested concentration. Primary human saphenous vein-derived SMCs were stimulated with PDGF-BB (50 ng/mL) and co-treated with hydroxytyrosol (HT; 10–320 µM) for 24 h or 48 h. Total nuclei (Hoechst^{+}, EdU^{+} and EdU^{-} combined) were quantified from the same fluorescence images used for EdU proliferation analysis (ten fields per condition per donor). Data are mean ± SEM from N = 6 independent donors, with individual donor means overlaid. Two-way ANOVA with Šídák's multiple comparisons test (each HT concentration and the untreated control vs. PDGF-BB alone) showed no significant difference in total nuclei count at any concentration or timepoint (all adjusted $p \geq 0.44$).

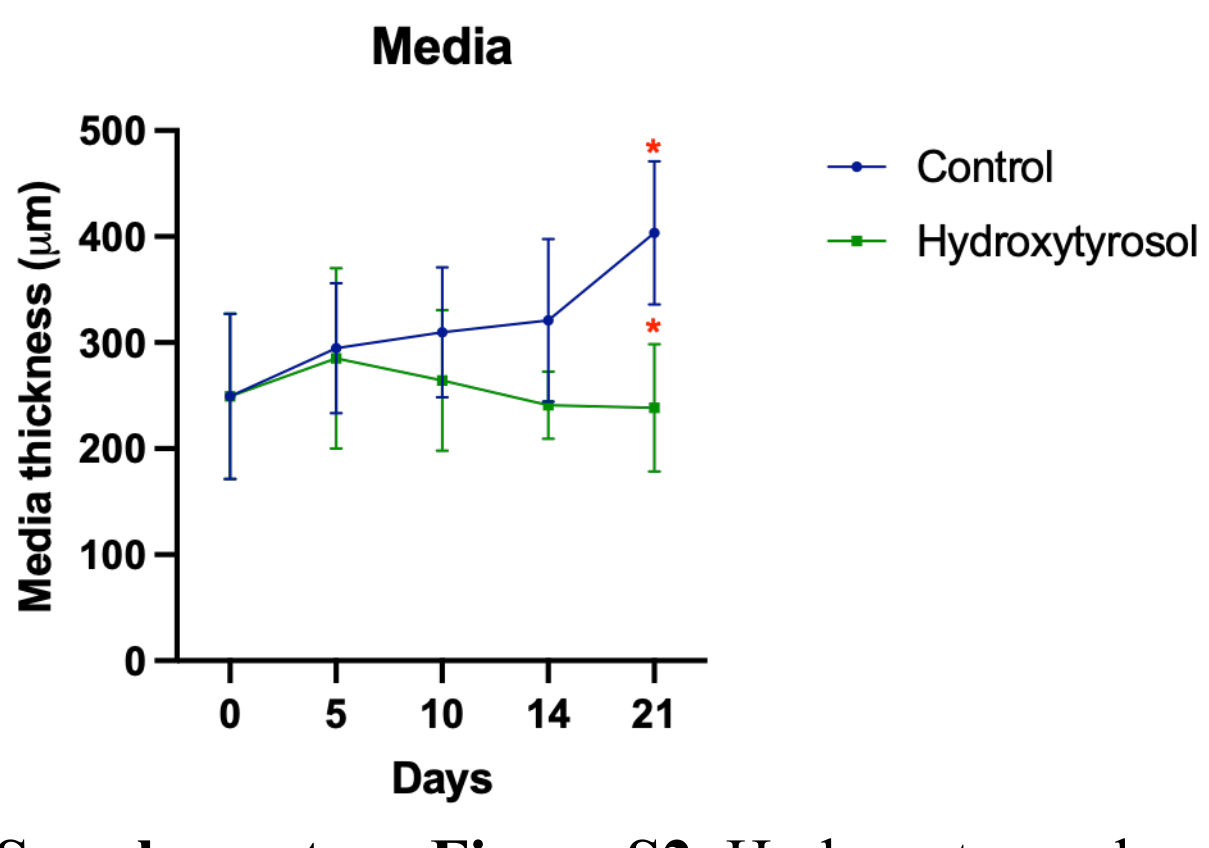

Supplementary Figure S2. Hydroxytyrosol reduces media thickness over the culture period. Media thickness (µm) was measured in control and hydroxytyrosol-treated cultures at days 0, 5, 10, 14, and 21. Data are mean ± SD, n = 5 per group per timepoint. Statistical significance was assessed by two-way ANOVA (treatment × time) with Tukey's multiple comparisons test. *$p < 0.05$ vs. control at the same timepoint.

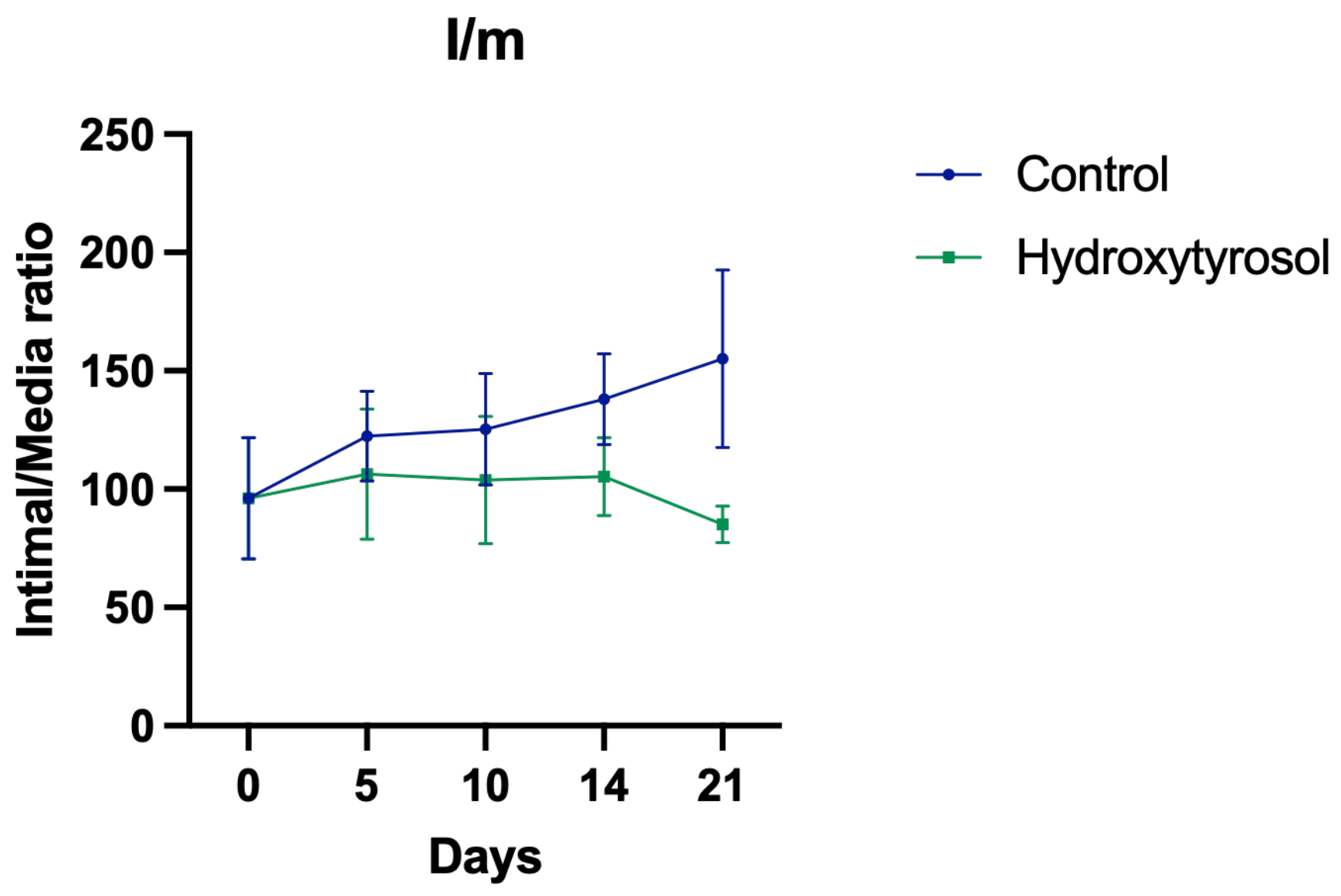

Supplementary Figure S3. Hydroxytyrosol does not alter the intima-to-media ratio. The intima/media (I/M) thickness ratio was calculated for each matched vessel at days 0, 5, 10, 14, and 21 in control and hydroxytyrosol-treated cultures. Data are mean ± SD, n = 5 per group per timepoint. Statistical significance was assessed by two-way ANOVA (treatment × time) with Tukey's multiple comparisons test; no significant differences were found between groups or across timepoints (all $p > 0.05$).
